# Genetic Insight Into Dissecting the FODMAP Diet Frame and Liver Cancer: The Mediation Role of Efferocytosis and Trogocytosis

**DOI:** 10.1002/fsn3.70883

**Published:** 2025-08-29

**Authors:** Xiang Ma, Kai Lei, Zuojin Liu

**Affiliations:** ^1^ Hepatobiliary Surgery The Second Affiliated Hospital of Chongqing Medical University Chongqing China

**Keywords:** efferocytosis, FODMAP diet, liver cancer, mendelian randomization, trogocytosis

## Abstract

The etiology of liver cancer remains poorly understood, particularly regarding its potential association with dietary patterns rich in fermentable oligosaccharides, disaccharides, monosaccharides, and polyols (FODMAP). This study investigated the genetic relationships between FODMAP‐related dietary intake and liver cancer risk and further assessed whether proteins involved in efferocytosis and trogocytosis mediate these associations. This study applied two‐sample Mendelian randomization (MR) and mediation MR analyses to examine the links among FODMAP‐related dietary factors, trogocytosis, efferocytosis, and liver cancer. Summary statistics were obtained from MVP‐GWAS for liver cancer, deCODE for trogocytosis and efferocytosis, and the UK Biobank for FODMAP dietary traits. A two‐step mediation MR analysis was then performed to determine whether the pathways of efferocytosis and trogocytosis mediated the genetic associations between FODMAP foods and liver cancer, with mediation proportions calculated accordingly. MR analyses revealed that higher consumption of cheese (OR = 0.548, 95% CI = 0.404–0.743), fresh fruit (OR = 0.375, 95% CI = 0.194–0.727), cereal (OR = 0.575, 95% CI = 0.386–0.857), and dried fruit (OR = 0.539, 95% CI = 0.340–0.853) was significantly associated with a reduced risk of liver cancer. Protein‐level analyses identified four trogocytosis‐ and efferocytosis‐related proteins, TGFB3, EPOR, ELANE, and C3, that may mediate these dietary effects on liver cancer susceptibility. Mediation MR indicated that cheese intake influenced liver cancer risk indirectly by modulating TGFB3, EPOR, ELANE, and C3 expression, accounting for 8.8%, 25%, 1.8%, and 12.7% of the total effect, respectively. Sensitivity analyses for heterogeneity and pleiotropy supported the robustness of these findings. This study uncovers a potential molecular mechanism by which FODMAP‐related dietary patterns may modulate liver cancer risk through the TGFB3/EPOR/ELANE/C3 signaling axis. These results provide genetic evidence and mechanistic insights supporting the role of FODMAP‐oriented dietary strategies in liver cancer prevention, offering a theoretical basis for future public health interventions.

## Graphical abstract

Liver cancer is a highly malignant cancer with a continuously rising global incidence. It is not clear whether a low fermentable oligosaccharides, disaccharides, monosaccharides, and polymers (FODMAP) diet frame is associated with LC. This study reveals the molecular mechanism by which the FODMAP diet regulates liver cancer progression through the Efferocytosis and Trogocytosis signaling axis. This study highlights the importance of the FODMAP Diet in the prevention and treatment of liver cancer.

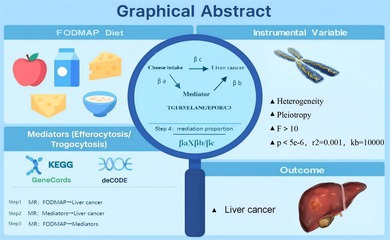

**Abbreviations**
C3: complement component 3
CI: confidence interval
ELANE: neutrophil elastase
EPOR: erythropoietin receptor
FFQ: food frequency questionnaire
FODMAP: fermentable oligosaccharides, disaccharides, monosaccharides, and polyols
GWAS: genome‐wide association studies
IV: instrumental variable
IVW: inverse‐variance weighted
LD: linkage disequilibrium
MR: Mendelian randomization
MVP: Million Veteran Program
OR: odds ratio
PCDs: programmed cell‐death
pQTL: protein quantitative trait locus
SCFA: short‐chain fatty acids
TGFB3: transforming growth factor beta 3

## 1 Introduction

Liver cancer is a highly aggressive malignancy with a steadily increasing global incidence and represents the fastest‐growing cause of cancer‐related mortality in China (Chen et al. 2020). Its insidious onset, characterized by nonspecific early symptoms, often results in delayed diagnosis and presentation at advanced stages, leaving patients with limited treatment options and poor survival outcomes (Brown et al. 2023). The pathogenesis of liver cancer is multifactorial, with well‐established risk factors such as chronic liver diseases, particularly cirrhosis secondary to viral hepatitis or chronic alcohol consumption, playing a major role (Schütte et al. 2012). Beyond these recognized determinants, diet has emerged as a modifiable exposure that may influence liver cancer susceptibility through microbiome‐dependent, metabolic, and immunological mechanisms (Tilg et al. 2023). However, the specific dietary components that causally affect liver cancer risk, and the underlying biological mechanisms, remain insufficiently defined.

The fermentable oligosaccharides, disaccharides, monosaccharides, and polyols (FODMAP) dietary framework offers a structured approach for investigating diet–cancer interactions (Janota and Szymanek 2024). Variations in FODMAP content across foods can modulate luminal fermentation, short‐chain fatty acid (SCFA) production, intestinal barrier integrity, bile acid signaling, and systemic inflammatory response pathways that are plausibly linked to hepatocarcinogenesis (Zhang et al. 2021; Yang et al. 2024). Observational studies have reported associations between consumption of dairy products, fruits, cereals, and dried fruits and liver cancer risk or intermediate hepatic phenotypes. However, such studies are inherently vulnerable to residual confounding, exposure misclassification, and reverse causation, limiting the strength of causal inferences. In light of the incomplete understanding of the molecular pathways connecting FODMAP intake to liver cancer, there is a pressing need for methodologically robust investigations to clarify these relationships.

Efferocytosis and trogocytosis, two specialized forms of programmed cell‐death processes (PCDs), are critical regulators of the tumor microenvironment. By influencing antigen presentation, cytokine profiles, and macrophage polarization, these processes may serve as conduits linking diet‐driven mucosal and metabolic signals to hepatic oncogenic or protective pathways (Chen et al. 2021; Cruz Cruz et al. 2023; Lecoultre et al. 2020). Emerging evidence suggests that the FODMAP diet may modulate these mechanisms, in part by attenuating SCFA‐mediated enhancement of efferocytic activity (Vieira et al. 2017). These observations raise the possibility that targeted modulation of dietary FODMAP content could represent a novel therapeutic strategy for liver cancer through precise regulation of cellular clearance dynamics.

Mendelian randomization (MR) leverages the random allocation of genetic variants during meiosis, functioning as a natural analog to randomized controlled trials (Rasooly and Patel 2019). Its primary methodological strength lies in its ability to minimize environmental confounding and reverse causation, common limitations of conventional observational designs (Koellinger and de Vlaming 2019). In this study, two‐sample MR was performed to evaluate the causal relationship between FODMAP‐related dietary intake and liver cancer risk, followed by a two‐step MR to investigate whether proteins involved in efferocytosis and trogocytosis, specifically TGFB3, EPOR, ELANE, and C3, mediate these effects. By establishing food‐specific causal effects and identifying plausible molecular mediators, this work aims to inform dietary guidelines for liver cancer prevention.

## 2 Methods

### 2.1 Study Design

**FIGURE 1:**
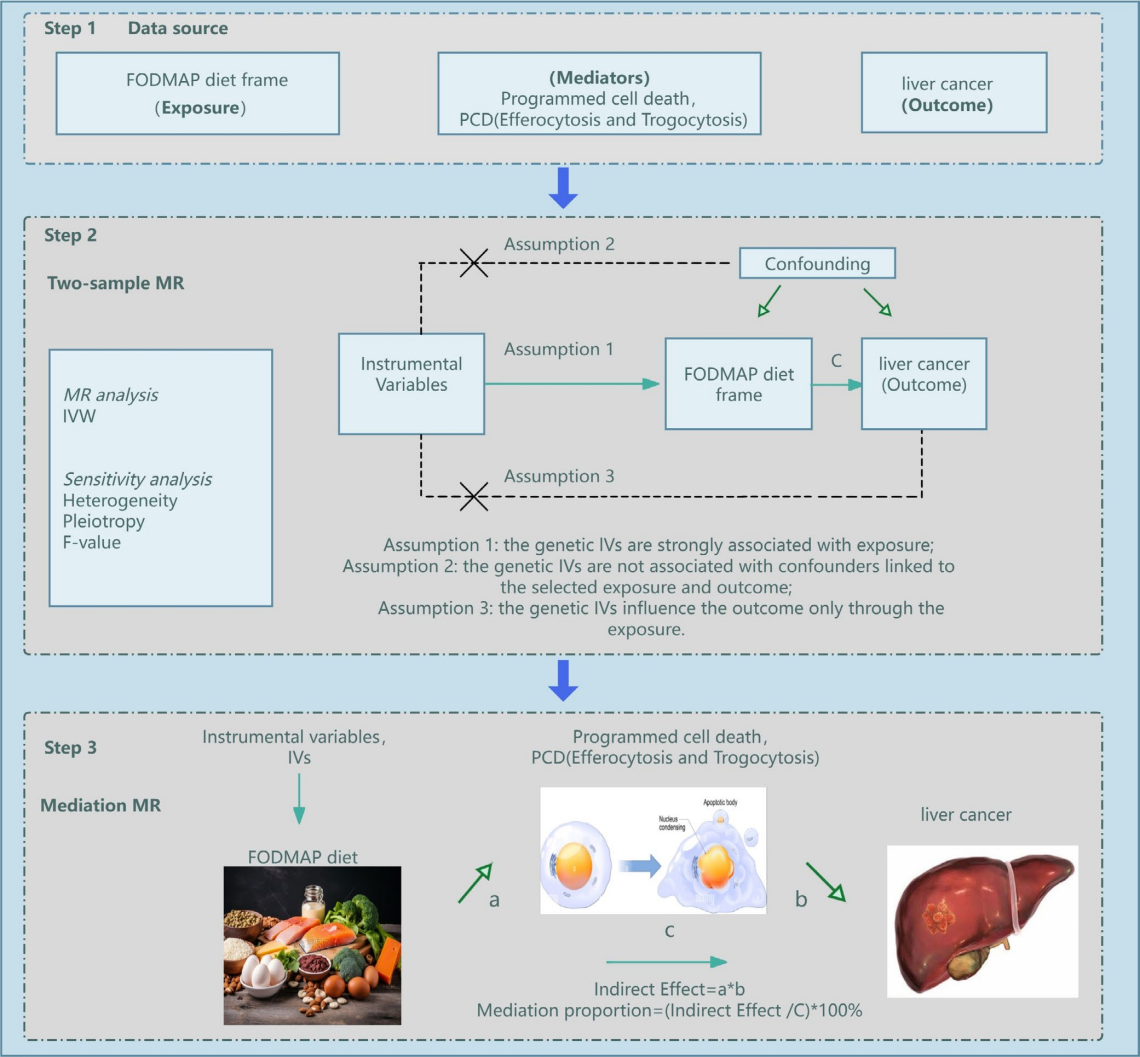
Schematic overview of the Mendelian randomization (MR) analysis framework used in this study. The flowchart illustrates the sequential steps undertaken: Exposures were FODMAP‐related food intakes, candidate mediators were proteins associated with efferocytosis and trogocytosis, and the outcome was liver cancer. First, the total effect of the FODMAP‐related diet on liver cancer risk was estimated. Next, we assessed the effect of each candidate protein on liver cancer, followed by evaluating the influence of diet on protein levels. Finally, the proportion of the diet–cancer association mediated by each protein was calculated.

This study was conducted following the *Strengthening the Reporting of Observational Studies in Epidemiology Using Mendelian Randomization* (STROBE‐MR) guidelines (see Supporting Informations for the STROBE‐MR checklist). The overall analytical workflow is illustrated in Figure 1. We employed publicly available multi‐omics datasets derived from ethically approved genome‐wide association studies (GWAS). Adhering to the three assumptions of MR, the present study first estimated the total effect of the FODMAP‐related dietary pattern on liver cancer risk, followed by the effect of each candidate protein on liver cancer, and subsequently the impact of diet on protein expression. Mediation proportions were then calculated accordingly. Ethical approval for this specific project was not required, as all analyses were based on publicly available GWAS summary statistics from previously published studies, with no use of individual‐level data.

### 2.2 Data Sources

This study employed genetic association data for 28 dietary factors obtained from a large‐scale GWAS conducted by the Medical Research Council Integrative Epidemiology Unit (MRC‐IEU) in 2018 (Skrivankova et al. 2021). The exposure dataset included foods classified as low‐FODMAP (e.g., bananas, broccoli, tomatoes, pork, beef, poultry, lamb, lobster/crab, fish, cheese, feta, bran cereal, tea, coffee, beer, and red wine), high‐FODMAP (e.g., apples, cherries, mangoes, garlic, celery, and milk), and other dietary items (e.g., fresh fruits, dried fruits, cooked vegetables, salads, and cereal). Details of these 28 FODMAP‐related dietary intakes are provided in Table S1. The dietary exposures were obtained from the IEU/OpenGWAS database, corresponding to food frequency questionnaire (FFQ)‐derived traits from the UK Biobank. Measurement units followed the original FFQ definitions, which reflected portions or frequencies rather than gram weights. Examples include pieces/day for fresh or dried fruit, bowls/week for cereal, heaped tablespoons/day for cooked vegetables, cups/day for tea or coffee, and frequency/week for categorical items such as cheese. The mapping of FFQ items to each dietary exposure is provided in Table S2. Further methodological details can be found in the UK Biobank Data Showcase (http://biobank.ndph.ox.ac.uk/showcase/).

For the two PCDs, efferocytosis and trogocytosis, we systematically identified associated genes through comprehensive searches of the KEGG database (https://www.genome.jp/kegg/) and GeneCards (https://www.genecards.org). After removing duplicate entries, we obtained 305 unique proteins related to efferocytosis and 27 genes related to trogocytosis (Table S3). Instrumental variables (IVs) for the proteins encoded by these proteins were subsequently retrieved from the deCODE database (Ferkingstad et al. 2021) (Table S3). Furthermore, inferences were made at the protein level, and the instruments for proteins involved in efferocytosis or trogocytosis were not interpreted as being exclusively pathway‐specific.

Liver cancer outcome data were obtained from the Department of Veterans Affairs (VA) Million Veteran Program (MVP), comprising 2852 cases and 447,587 controls of European ancestry (Verma et al. 2024). Comprehensive clinical phenotype information was extracted from the VA Electronic Health Record system, including diagnostic codes, longitudinal laboratory test results, and serial vital sign measurements. These rigorous data collection procedures ensured high‐quality and reliable outcome data for analysis (Table S1).

### 2.3 Genetic Instrumental Variable Selection

The present study employed a comprehensive screening strategy to select instrumental genetic variants, aiming to balance instrument strength with sufficient quantity. Single‐nucleotide polymorphisms (SNPs) associated with the FODMAP diet were identified using a significance threshold of *p* < 5 × 10^−6^. All candidate SNPs were subjected to stringent quality control procedures, beginning with linkage disequilibrium (LD) pruning at an *R* ^2^ threshold of < 0.001 within a 10,000 kb window to ensure genetic independence. Effect alleles were then carefully harmonized between exposure and outcome datasets to preserve directional consistency in effect estimates. Instrument strength was quantitatively evaluated using F‐statistics, applying a conservative threshold of *F* > 10 to minimize the risk of weak instrument bias. For pQTL selection, we used rigorous criteria: (1) only genome‐wide significant associations (*p* < 5 × 10^−8^) were retained; (2) variants within the major histocompatibility complex (MHC) region (chromosome 6: 26–34 Mb) were excluded; (3) independence was ensured through LD‐based clumping (*R* ^2^ < 0.1 within a 10,000 kb window); and (4) only cis‐pQTLs, located within ±1 Mb of their corresponding target gene, were included.

### 2.4 Mendelian Randomization

This study utilized a two‐sample MR framework to systematically assess the causal relationships among the FODMAP diet, efferocytosis, trogocytosis, and liver cancer. The inverse‐variance weighted (IVW) method served as the primary analytical approach, complemented by a series of sensitivity analyses to evaluate the robustness of the findings. Cochran's Q test was used to assess potential heterogeneity among the genetic instruments (Kulinskaya and Dollinger 2015), with *p*_heterogeneity_ > 0.05 indicating no significant heterogeneity. MR‐Egger regression was then applied to determine horizontal pleiotropy (Wang et al. 2023), where *p*_pleiotropy_ > 0.05 suggested the absence of pleiotropic effects. MR‐PRESSO analysis was conducted to detect and, where possible, correct for horizontal pleiotropy by identifying and removing significant outlier variants. All MR analyses were performed using the TwoSampleMR package (version 0.6.8).

### 2.5 Mediation Analysis

**FIGURE 2:**
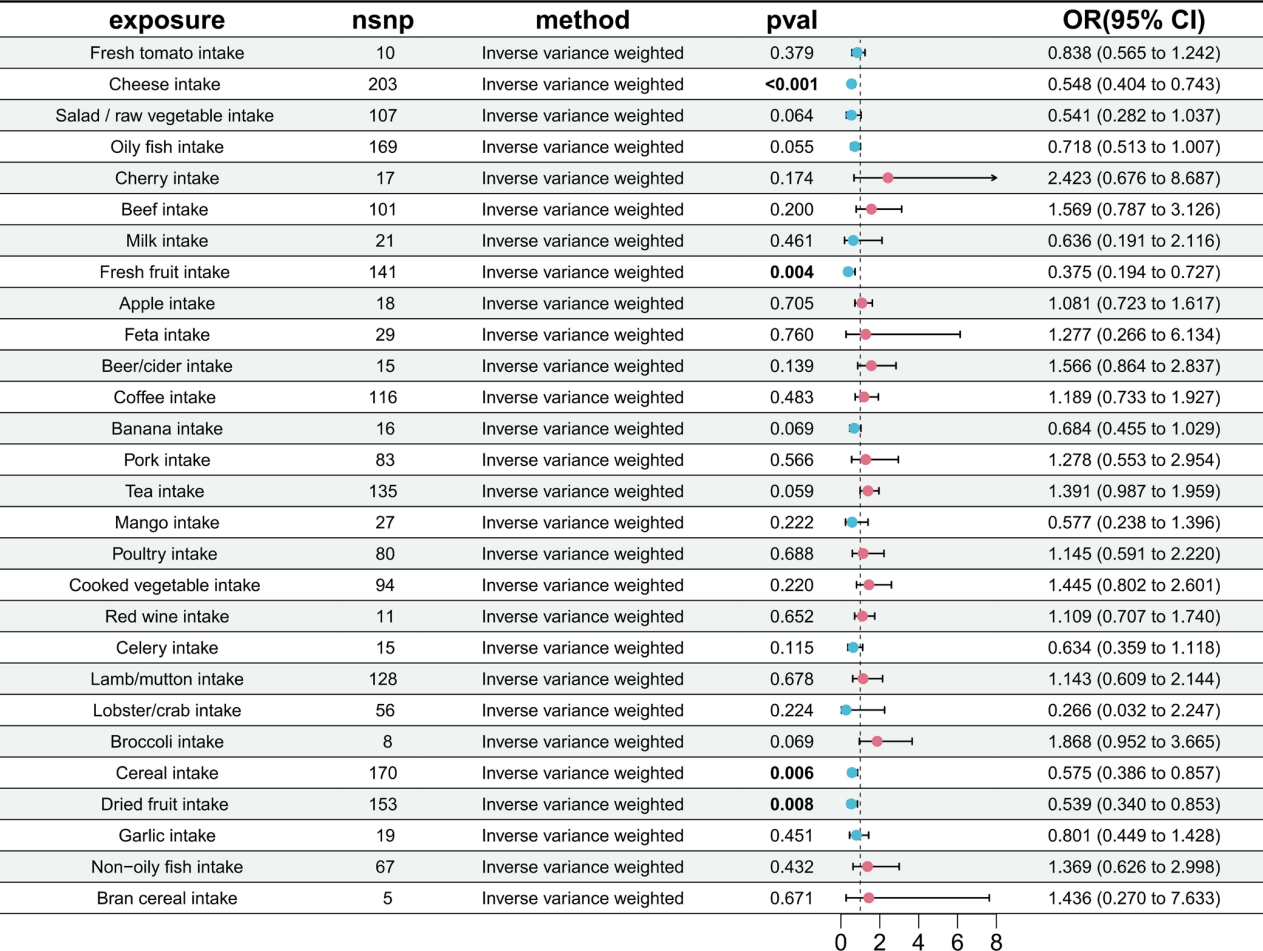
Causal associations between FODMAP‐related food intake and liver cancer risk. Forest plot displaying MR estimates for each FODMAP‐related food. For each row, the number of SNP instruments (*n*_SNP_), primary estimator (IVW), *p*‐value, and odds ratio (OR) with 95% confidence interval (CI) per 1‐standard deviation increase in genetically predicted intake are presented. OR values below 1 indicate a reduced liver cancer risk. Statistically significant associations (*p* < 0.05) are highlighted.

Mediation analysis was performed to evaluate whether the relationship between the FODMAP diet and liver cancer risk was mediated through efferocytosis and trogocytosis pathways. The total effect of the FODMAP diet on liver cancer comprised two components: (1) the direct effect of the FODMAP diet on liver cancer risk (pathway *c* in Figures 1 and 2) the indirect effect mediated by efferocytosis and trogocytosis (pathway *a* × *b* in Figure 1). The proportion of the indirect effect relative to the total effect was used to quantify the potential mediating role of these pathways.

Given that mediation analysis assumes the absence of unmeasured confounding, we employed genetic instruments to minimize confounding bias and conducted sensitivity analyses to assess the robustness of the results.

## 3 Results

### 3.1 The Associations of FODMAP Diet With Liver Cancer

To evaluate the strength of each instrumental variable, we calculated the F‐statistics for the association between each instrument and its corresponding exposure. In all cases, the *F*‐statistics were well above the threshold of 10, indicating that the selected SNPs were strong instruments (Tables S4 and S5).

As shown in Figure 2, IVW analyses identified four dietary factors, cheese, fresh fruit, cereal, and dried fruit, that were inversely associated with liver cancer, with all ORs below 1 (Figure 2 and Table S6). The IVW method demonstrated significant protective associations for cheese intake (OR = 0.548, 95% CI: 0.404–0.743, *p* = 1.06 × 10^−4^), fresh fruit consumption (OR = 0.375, 95% CI: 0.194–0.727, *p* = 3.66 × 10^−6^), cereal intake (OR = 0.575, 95% CI: 0.386–0.857, *p* = 6.49 × 10^−3^), and dried fruit consumption (OR = 0.539, 95% CI: 0.340–0.853, *p* = 8.6 × 10^−3^).

Heterogeneity tests revealed heterogeneity in liver cancer for 10 diets: Cheese intake (IVW: *p*_heterogeneity_ = 0.009), Beef intake (IVW: *p*_heterogeneity_ = 0.001), Milk intake (*p*_heterogeneity_ = 0.024), Fresh fruit intake (IVW: *p*_heterogeneity_ = 0.003), Beer/cider intake (IVW: *p*_heterogeneity_ = 0.01), Coffee intake (IVW: *p*_heterogeneity_ = 0.006), Pork intake (IVW: *p*_heterogeneity_ = 0.001), Poultry intake (IVW: *p*_heterogeneity_ = 0.046), Lamb/mutton intake (IVW: *p*_heterogeneity_ = 0.001), Non‐oily fish intake (IVW: *p*_heterogeneity_ = 0.031) (Table S6). In the MR‐Egger intercept test, we detected no significant evidence of horizontal pleiotropy (*p*_pleiotropy_ > 0.05) (Table S6).

Four dietary categories, cheese, fresh fruit, cereal, and dried fruit, were significantly associated with liver cancer risk. All odds ratios were < 1, indicating that a genetically predicted 1‐unit increase in the intake of each food was linked to a reduced risk of liver cancer. These foods may influence hepatic immune regulation by modulating macrophage efferocytosis and complement activity. Diets high in fiber are known to increase SCFAs, antioxidants, and polyphenols. In comparison, fermentable fiber‐rich diets specifically alter SCFA production and bile acid signaling mechanistic pathways that align with our identified mediation targets.

### 3.2 The Associations Between Efferocytosis, Trogocytosis, and Liver Cancer

**FIGURE 3:**
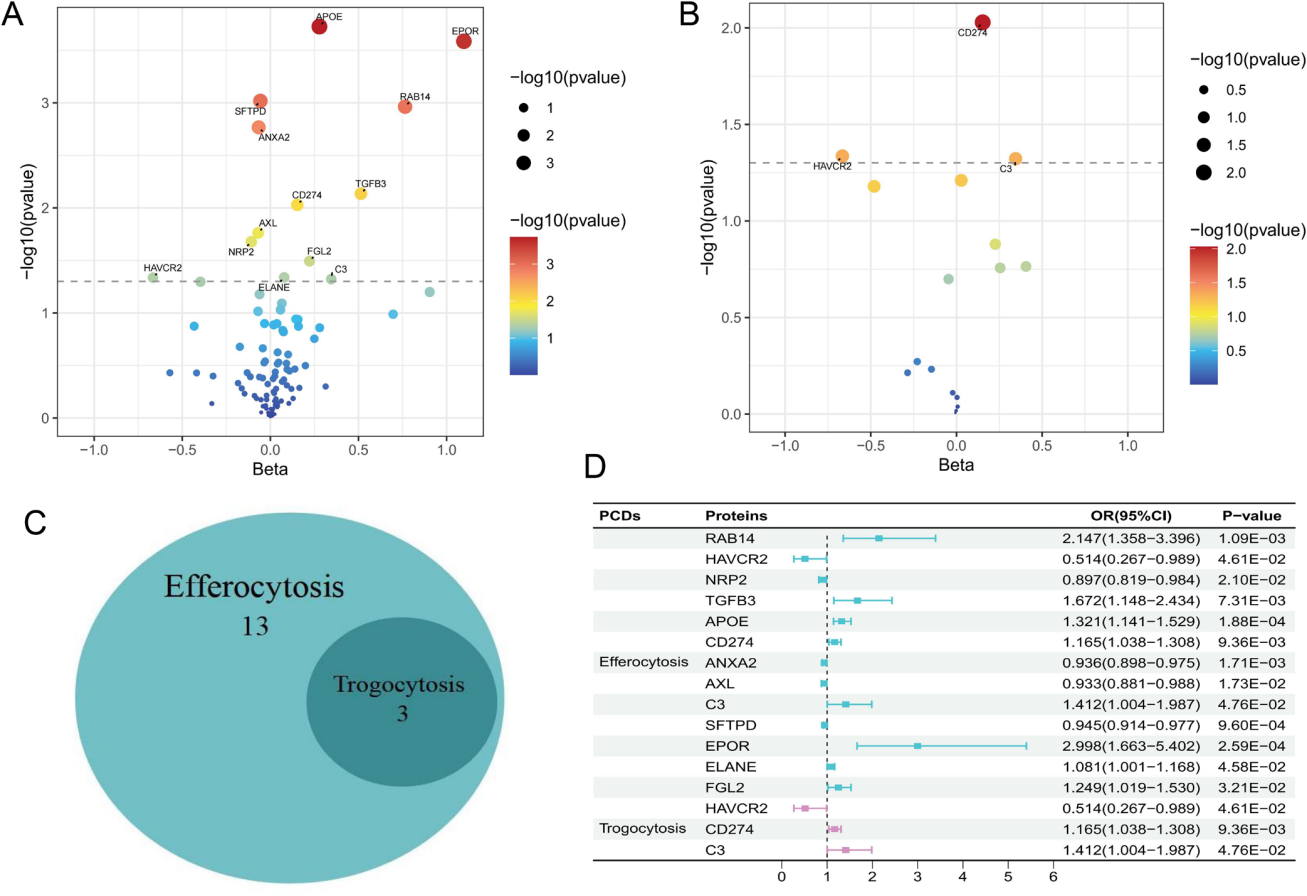
Selection of efferocytosis/trogocytosis‐related proteins and their associations with liver cancer. (A, B) Volcano plots showing standardized effect estimates (β; *x*‐axis) versus statistical significance (−log_10_ *p*‐value; *y*‐axis) from screening analyses. Point size and color reflect *p*‐value magnitude, and labeled markers highlight key proteins of interest (e.g., TGFB3, EPOR, ELANE, C3). The dashed horizontal line denotes the nominal significance threshold (*p* < 0.05). (C) Venn diagram illustrating the overlap in proteins mapped to efferocytosis and trogocytosis pathways. (D) Forest plot of MR estimates for the association between each protein and liver cancer, where OR > 1 indicates increased risk and OR < 1 indicates decreased risk.

Mendelian randomization was performed to examine the causal roles of efferocytosis and trogocytosis in liver cancer risk. Figure 3 presents the workflow for protein screening and prioritization. In the MR analysis of 116 efferocytosis‐related pQTLs, 13 proteins were identified as being causally associated with liver cancer risk. Eight proteins showed a positive association with risk: APOE (OR = 1.321, 95% CI: 1.141–1.529, *p* = 1.88 × 10^−4^), RAB14 (OR = 2.147, 95% CI: 1.358–3.396, *p* = 1.09 × 10^−3^), ELANE (OR = 1.081, 95% CI: 1.001–1.168, *p* = 4.58 × 10^−2^), TGFB3 (OR = 1.672, 95% CI: 1.148–2.434, *p* = 7.31 × 10^−3^), C3 (OR = 1.412, 95% CI: 1.004–1.987, *p* = 4.76 × 10^−2^), CD274 (OR = 1.165, 95% CI: 1.038–1.308, *p* = 9.36 × 10^−3^), FGL2 (OR = 1.249, 95% CI: 1.019–1.530, *p* = 3.21 × 10^−2^), and EPOR (OR = 2.998, 95% CI: 1.663–5.402, *p* = 2.59 × 10^−4^).

In comparison, five proteins demonstrated a protective association: NRP2 (OR = 0.897, 95% CI: 0.819–0.984, *p* = 2.10 × 10^−2^), ANXA2 (OR = 0.936, 95% CI: 0.898–0.975, *p* = 1.71 × 10^−3^), SFTPD (OR = 0.945, 95% CI: 0.914–0.977, *p* = 9.60 × 10^−4^), HAVCR2 (OR = 0.514, 95% CI: 0.267–0.989, *p* = 4.61 × 10^−2^), and AXL (OR = 0.933, 95% CI: 0.881–0.988, *p* = 1.73 × 10^−2^) (Figure 3A).

Analysis of 21 trogocytosis‐related proteins identified three with causal associations to liver cancer: HAVCR2, CD274, and C3 (Figure 3B). These three genes were also shared with the efferocytosis pathway, as shown in the Venn diagram (Figure 3C,D, Table S7). Sensitivity analyses revealed evidence of between‐variant heterogeneity but found no indication of directional horizontal pleiotropy based on pleiotropy‐robust diagnostics (Table S7).

Overall, proteins involved in immune checkpoint regulation, complement activation, neutrophil protease activity, lipid and vesicular trafficking, and growth factor signaling were associated with an increased risk of liver cancer. In comparison, proteins with protective associations were linked to the maintenance of innate barrier integrity and clearance homeostasis.

### 3.3 Six Proteins as Potential Intervention Targets Modulated by the FODMAP Diet

**FIGURE 4:**
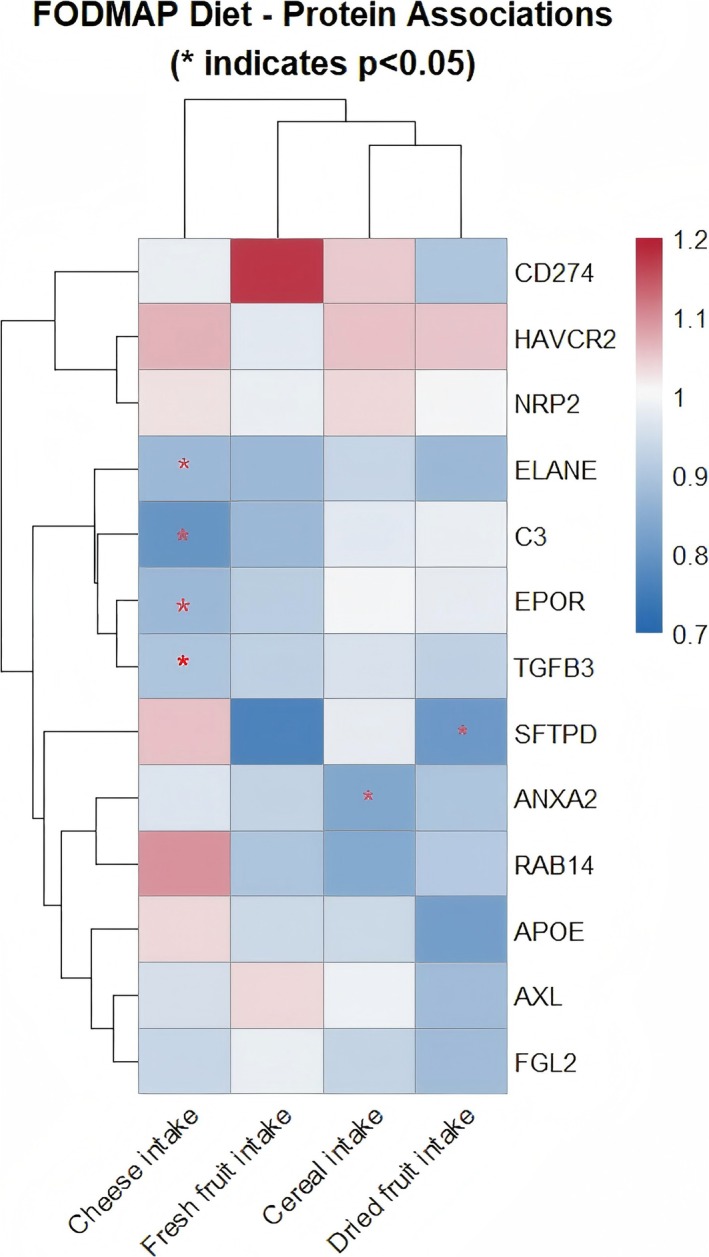
Heatmap illustrating the associations between FODMAP‐related foods and efferocytosis/trogocytosis proteins. Columns represent the four food types, whereas rows list the candidate proteins. Asterisks indicate nominal statistical significance (*p* < 0.05).

After establishing the associations between the FODMAP dietary pattern and liver cancer, and identifying liver cancer‐related proteins, we performed MR analyses to explore potential links between FODMAP diet components and these proteins. As shown in Figure 4, the MR results revealed significant associations between three FODMAP dietary components, cheese, cereal, and dried fruit intake, and six liver cancer‐related proteins (ANXA2, SFTPD, TGFB3, EPOR, ELANE, and C3) (Figure 4; Table S8).

Cheese, in particular, provides calcium and bioactive peptides, and modulates bile acid composition and colonic fermentation processes known to influence TGF‐β (TGFB3) signaling, complement (C3) activity, and macrophage clearance pathways. These findings align with biologically plausible mechanisms along the gut–liver axis.

### 3.4 Cheese Intake Mediates the FODMAP Diet's Effect on Liver Cancer Risk

**FIGURE 5:**
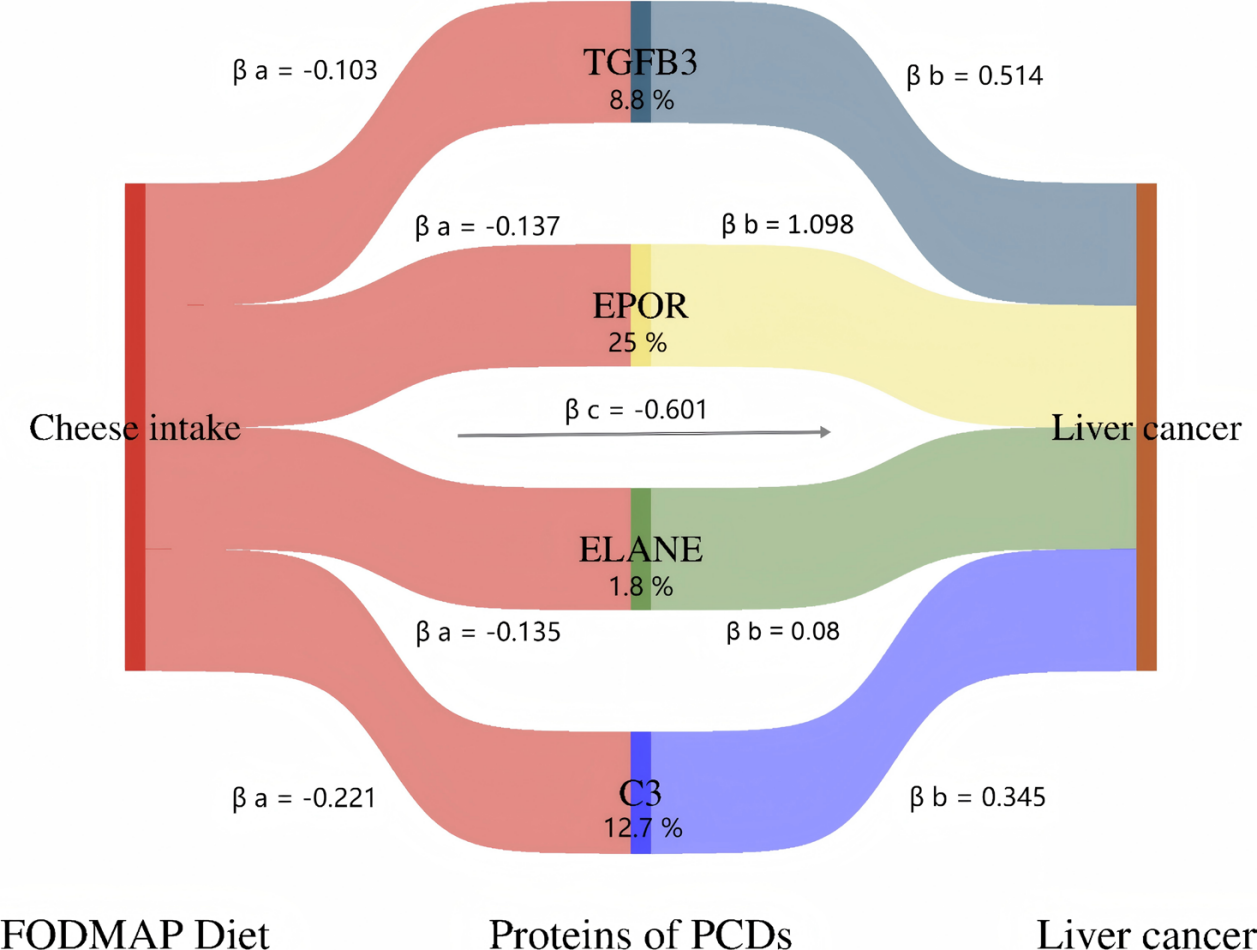
Mediation model illustrating how the FODMAP‐related diet influences liver cancer risk through programmed cell‐clearance pathways. Path *a*: Cheese → protein; path *b*: Protein → liver cancer. The total effect represents the direct association between cheese intake and liver cancer, while the indirect effect is calculated as *a* × *b*. Estimated mediation proportions: TGFB3 (8.8%), EPOR (25.0%), ELANE (1.8%), and C3 (12.7%).

Figure 5 presents the FODMAP diet–protein–liver cancer relationships. Using a two‐step MR framework, we evaluated whether TGFB3, EPOR, ELANE, and C3 mediate the causal association between FODMAP dietary intake and liver cancer risk. We estimated: (1) the total effect (βc: cheese intake on liver cancer), (2) the indirect effect (βa × βb, where βa is the effect of cheese intake on each protein, and βb is the effect of that protein on liver cancer), and (3) the direct effect (βc′ = βc − βa × βb) (Figure 1; Table 1). MR findings suggest that higher cheese intake is associated with reduced liver cancer risk, with partial mediation through modulation of TGFB3, EPOR, ELANE, and C3 expression. These proteins accounted for 8.8%, 25.0%, 1.8%, and 12.7% of the total effect, respectively.

**TABLE 1:** Mediator analysis results of the FODMAP diet, PCDs protein expression, and liver cancer disease risk.

FODMAP diet	Proteins of PCDS	Path a, βa	Path b, βb	Total effect, βc	Direct eiffect, βc	βa*b	Proportion mediated (%)
Cheese intake	TGFB3	−0.103	0.514	−0.601	−0.548	−0.053	0.088
	EPOR	−0.137	1.098	−0.601	−0.451	−0.15	0.25
	ELANE	−0.135	0.08	−0.601	−0.59	−0.0108	0.018
	C3	−0.221	0.345	−0.601	−0.525	−0.076	0.127

The protective influence of cheese intake may act, at least in part, by dampening immunosuppressive and inflammatory pathways, potentially via microbiome‐derived metabolites, maintenance of barrier integrity, or enhancement of clearance mechanisms.

## 4 Discussion

The findings of the present study indicate that higher consumption of cheese, fresh fruits, cereals, and dried fruits is significantly associated with a reduced risk of liver cancer. This highlights the potential role of specific dietary components, particularly certain FODMAP‐related foods, in liver cancer prevention, and provides genetic evidence linking these effects to efferocytosis‐ and trogocytosis‐related proteins.

Cheese may exert chemopreventive effects through multiple mechanisms, including calcium and bioactive peptide delivery, modulation of gut microbiota, enhancement of SCFA production, and suppression of oxidative stress and inflammatory signaling. Its bioactive components, particularly calcium and probiotics, have been shown to confer protection against various malignancies, including liver cancer (Molska and Reguła 2019). The calcium in cheese shows protective effects against colorectal cancer (Barrubés et al. 2019), and this benefit may extend to liver cancer through shared metabolic pathways (Zhao et al. 2021). Furthermore, the probiotic content in fermented dairy products modulates gut microbiota composition (Kamil et al. 2022), potentially improving hepatic metabolism and reducing carcinogenic processes (Huang et al. 2019; Li et al. 2014; Qiao et al. 2022). Fresh fruits supply vitamins, minerals, fiber, and polyphenols with potent antioxidant and anti‐inflammatory properties. These bioactive compounds, particularly flavonoids and polyphenols, can attenuate oxidative stress and inflammatory responses, two central drivers of hepatocarcinogenesis. Epidemiological evidence consistently links higher fruit intake with decreased liver cancer incidence (Yang et al. 2020), attributable primarily to their abundant flavonoid and polyphenol content (Li et al. 2023). These phytochemicals demonstrate remarkable anti‐inflammatory and antioxidant capacities, effectively mitigating oxidative stress and inflammatory responses—two fundamental pathological drivers of liver cancer (Holt et al. 2009; Wang et al. 2016). Whole grain cereals and dried fruits similarly contribute to hepatoprotective dietary patterns. The high dietary fiber content in whole grains has been inversely correlated with cancer risk across multiple organ systems, including the liver (Liu et al. 2021). Cereal fiber strengthens intestinal barrier integrity, promotes fermentation to short‐chain fatty acids, and increases stool bulk and transit, diluting and eliminating potential carcinogens (Liikonen et al. 2024). Similarly, dried fruits, particularly those rich in antioxidants, offer protection by scavenging free radicals and minimizing oxidative damage to hepatocytes (Kamiloglu et al. 2016; Turan and Celik 2016).

Furthermore, these findings emphasize the pivotal roles of TGFB3, EPOR, and ELANE in liver cancer, especially regarding dietary factors such as cheese intake. TGFB3, a member of the transforming growth factor‐beta superfamily, is recognized for its diverse functions in regulating cellular proliferation, differentiation, and apoptosis (Wu et al. 2005). Studies have indicated that TGFB3 can modulate immune responses and tissue repair mechanisms (Nolte and Margadant 2020), which are critical in the tumor microenvironment (Nolte and Margadant 2020). The inverse association between cheese consumption and liver cancer risk may be partly explained by TGFB3's capacity to modulate inflammatory pathways, potentially suppressing tumorigenesis. These mediators TGFB3, EPOR, ELANE, and C3 highlight four potential mechanistic routes. Given that MR analyses use genetic proxies for intake, the observed direction of effect is likely causal; however, experimental studies are needed to validate these findings.

EPOR, the erythropoietin receptor, is best known for its role in erythropoiesis (Tsiftsoglou 2021); however, accumulating evidence points to its involvement in cancer biology. EPOR signaling can improve cell survival and proliferation across various malignancies, and our Mendelian randomization findings suggest that genetic variation affecting EPOR‐related protein levels may contribute to the pathway linking certain FODMAP‐related foods, such as cheese, to liver cancer risk (Zhang et al. 2022). The present mechanistic analyses highlight EPOR as a potential key mediator connecting FODMAP dietary components with liver cancer development.

ELANE, also known as neutrophil elastase, is a serine protease integral to immune defense and has been implicated in numerous inflammatory disorders and cancers (Li et al. 2022). Elevated ELANE levels are often linked to poor cancer prognosis. Recent mechanistic studies have shown that ELANE promotes KEAP1 protein stability, inhibiting NRF2‐mediated ferroptosis in metabolic dysfunction–associated steatotic liver disease. These insights align closely with our finding that ELANE may mediate the relationship between cheese consumption and liver cancer risk.

Complement component C3 has emerged as a pivotal player in immune regulation and is implicated in a wide range of pathological conditions, including cancer. As a central element of the complement system, C3 is essential for opsonization (Erdei et al. 1991), inflammation, and the clearance of apoptotic cells. Research highlights its dual role in tumor biology, with the capacity to either promote or inhibit tumor progression depending on the context (Koumenis et al. 2014). In liver cancer, accumulating evidence suggests that C3 influences disease development by modulating the tumor microenvironment and shaping immune cell infiltration. These findings further indicate that C3 may mediate the protective association between cheese consumption and liver cancer risk, offering a strong rationale for investigating its potential as a therapeutic target.

In summary, this study demonstrates that certain protective foods, particularly cheese, are partly associated with reduced liver cancer risk through the modulation of TGFB3, EPOR, ELANE, and C3, suggesting that dietary patterns may influence liver cancer susceptibility by regulating cell‐clearance processes and innate immune signaling along the gut–liver axis. This study provides the causal evidence linking FODMAP dietary patterns to liver cancer risk using Mendelian randomization, while also identifying specific programmed cell death–related genes mediating this relationship, offering fresh perspectives for liver cancer prevention strategies. The methodological rigor of the present approach is significant. By applying robust IVW estimation methods alongside extensive sensitivity analyses, statistically reliable findings were generated that account for potential pleiotropic confounding, reinforcing the credibility of our causal inferences.

However, several limitations warrant further consideration. Although we have characterized the role of trogocytosis and efferocytosis pathways, the broader spectrum of programmed cell death mechanisms in hepatocarcinogenesis remains incompletely defined. Moreover, given the disproportionate global burden of liver cancer and the limited ethnic diversity in current datasets, we are actively establishing international collaborations to validate our results across racially and ethnically diverse populations through expanded data access and prospective multicenter studies. Finally, it should also be noted that pathway classifications are approximate, and certain variants may exert effects through alternative biological mechanisms. Thus, we interpret mediation at the protein level without claiming exclusivity to efferocytosis or trogocytosis.

## 5 Conclusions

In conclusion, this study demonstrates that FODMAP‐related dietary patterns are linked to liver cancer risk. Higher genetically predicted intakes of cheese (OR = 0.548, 95% CI = 0.404–0.743), fresh fruit (OR = 0.375, 95% CI = 0.194–0.727), cereal (OR = 0.575, 95% CI = 0.386–0.857), and dried fruit (OR = 0.539, 95% CI = 0.340–0.853) were each associated with a lower risk of liver cancer. Two‐step MR analyses further revealed that proteins involved in efferocytosis/trogocytosis partially mediate these associations, with estimated mediated proportions of 8.8% for TGFB3, 25.0% for EPOR, 1.8% for ELANE, and 12.7% for C3. These results implicate cell‐clearance pathways along the gut–liver axis as a biologically plausible link between diet and liver carcinogenesis. They also offer quantitative evidence to support dietary recommendations and public health strategies for liver cancer prevention, while identifying mechanistic targets for future clinical trials and population‐level interventions.

## Author Contributions

**Xiang Ma:** methodology (equal), writing – original draft (equal). **Kai Lei:** software (equal), visualization (equal). **Zuojin Liu:** investigation (equal), methodology (equal).

## Ethics Statement

The authors have nothing to report.

## Consent

The authors have nothing to report.

## Conflicts of Interest

The authors declare no conflicts of interest.

## Supporting information


**Tables S1–S8:** fsn370883‐sup‐0001‐TableS1‐S8.xlsx.

**Data S1:** fsn370883‐sup‐0002‐DataS1.docx.

## Data Availability

The GWAS summary statistics for pQTLs are available in the deCODE (https://www.decode.com/summarydata/). The GWAS summary data of liver cancer are available in published research (Verma et al. 2024). The GWAS summary data of deet are available at the IEU OPEN GWAS (IEU OpenGWAS project).
